# Clinical trial awareness and participation attitudes among patients with epidermolysis bullosa and their caregivers: a nationwide cross-sectional study from Saudi Arabia

**DOI:** 10.3389/fmed.2026.1913338

**Published:** 2026-09-03

**Authors:** Ashjan Alheggi, Saifaleslam A. Mahmoud, Danah Alali, Abrar Bukhari, Salma Albargawi, Rayan Alkhodair, Christine Wagger, Sophie Kitzmueller

**Affiliations:** 1 Department of Dermatology, College of Medicine, Imam Mohammad Ibn Saud Islamic University (IMSIU), Riyadh, Saudi Arabia; 2 General Medicine Practice Program, Batterjee Medical College, Aseer, Saudi Arabia; 3 General Practitioner, King Faisal Hospital, Al-Ahasa, Saudi Arabia; 4 Division of Pediatric Dermatology, Department of Pediatrics, King Abdullah Specialized Children’s Hospital, Riyadh, Saudi Arabia; 5 College of Medicine, King Saud Bin Abdulaziz University for Health Sciences, Riyadh, Saudi Arabia; 6 Research Center, King Abdullah International Medical Research Center, Riyadh, Saudi Arabia; 7 Department of Dermatology and Allergology and EB House Austria, University Hospital of the Paracelsus Medical University, Salzburg, Austria

**Keywords:** clinical trial, epidermolysis bullosa, motivators, patient perspective, rare disease

## Abstract

**Background:**

Epidermolysis bullosa (EB) is a rare, inherited skin fragility disorder with significant disease burden and rapidly evolving therapeutic options. Clinical trial recruitment in rare diseases remains challenging; understanding patient perspectives is essential to designing patient-centred protocols. The objective of this study was to explore clinical trial awareness, motivators, and barriers among patients with EB and their caregivers in Saudi Arabia.

**Materials and methods:**

This cross-sectional study recruited participants of all EB subtypes and ages from the Saudi EB Registry. A 40-item structured, self-administered questionnaire assessed clinical background, knowledge and attitudes toward clinical trials, motivators and barriers for participation. Responses were recorded on a five-point Likert scale. Data were analysed using IBM SPSS Statistics version 23.

**Results:**

Thirty-five participants were enrolled; 68.6% were younger than 18 years and 57.1% of questionnaires were completed by caregivers. Knowledge about EB clinical trials was low in 80% of participants; the level of knowledge was statistically significantly associated with the desire to participate (*p* = 0.001). Despite low knowledge, willingness to participate was high or very high in 51.4% of participants. Practical motivators ranked highest; travel and accommodation cost coverage and better medical care during the study period were rated as highly important by 94.3% each. Altruistic motivators followed closely; contributing to increased disease knowledge and future improvements for other patients with EB were highly important to 91.4% and 88.6% respectively. The dominant barriers were distance and travel costs and concern about being in the placebo group (65.7% each), followed by lack of clear information about the study protocol (62.9%).

**Conclusions:**

This study provides the characterisation of clinical trial awareness, motivators, and barriers among patients with EB and their caregivers. Patient education is the most actionable target for improving trial recruitment. The high prioritisation of symptom alleviation underscores the importance of incorporating patient-reported outcome measures as primary or co-primary endpoints in future EB trial design. Trial protocols in EB should minimise travel burden and invasive procedures, address paediatric-specific barriers including route of administration, frequency, and school absenteeism, and incorporate flexible, patient-centred designs to optimise enrolment across the EB community.

## Introduction

Epidermolysis bullosa (EB) is a rare, heterogeneous group of inherited genodermatoses characterised by hyperfragility of epithelial tissues and blister formation following minimal mechanical trauma (1). The disease is classified into four major subtypes based on the level of skin cleavage and the underlying genetic mutation: EB simplex (EBS), junctional EB (JEB), dystrophic EB (DEB), and Kindler EB (2). Severe subtypes, particularly recessive DEB (RDEB) and JEB, carry high morbidity and mortality; chronic wounds, mucosal involvement, and multi-organ complications define their clinical course (3).

Current management is multidisciplinary and largely symptomatic; wound care, nutritional support, pain control, and complication management constitute the mainstay of treatment (4). The therapeutic landscape has evolved rapidly; beremagene geperpavec (Vyjuvek), the first topical gene therapy for DEB, *utilises a replication-incompetent herpes simplex virus type 1 vector to restore COL7A1 expression at the wound site and received FDA approval in 2023* (5). Birch triterpenes (Filsuvez), *a topical gel derived from birch bark extract with wound-healing properties, received approval* for DEB and JEB wounds *management* (6). Most recently prademagene zamikeracel (Zevaskyn), the first cell-based gene therapy for RDEB, *employs retrovirally corrected autologous keratinocyte sheets to restore type VII collagen expression and received FDA approval in 2025* (7). *Together, these advances represent a paradigm shift in EB management and underscore the critical importance of robust clinical trial infrastructure to support future therapeutic development* (5–7). Clinical trials are therefore an indispensable pathway to advancing EB care; yet rare disease trials face inherent challenges: small patient populations, geographic dispersion, recruitment failures, and high trial burden (8).

Patient and caregiver perspectives are critical determinants of trial recruitment and retention (9). Understanding the motivators, barriers, and level of knowledge about clinical trials enables the design of patient-centred protocols that improve enrolment and reduce dropout rates (10, 11).

This study aimed to assess the level of knowledge, motivators, and barriers to clinical trial participation among patients with EB and their caregivers in Saudi Arabia, and to identify factors associated with willingness to participate. The findings provide the first nationwide characterisation of clinical trial perspectives in this population and offer actionable guidance for designing patient-centred EB trials.

## Materials and methods

### Study design and participants

This cross-sectional study evaluated clinical trial awareness and participation attitudes among patients with EB and their caregivers in Saudi Arabia. Participants were recruited from the Saudi EB Registry. Eligible participants had a confirmed diagnosis of EB irrespective of subtype or age, identified Arabic as their native language, and provided written informed consent.

### Questionnaire

A 40-item structured, self-administered questionnaire was used for data collection. It was modified with permission from Prodinger et al., whose original instrument comprised 53 items across six sections evaluating knowledge and attitudes towards clinical trial participation among patients with EB (12).

The questionnaire comprised five sections: demographics (6 items), general clinical background (2 items), knowledge and information about clinical studies (7 items), motivators for participation (11 items), and barriers to participation (14 items). Modifications aimed to improve contextual relevance for the Saudi population with EB while preserving the instrument's core domains.

Four items specific to the EB House Austria were removed; these assessed institutional quality of life, general health status, and medical care. The entire expenses section (7 items) was also removed; no large-scale EB clinical trial centres currently operate in Saudi Arabia, rendering those items inapplicable.

The demographics section was expanded from four to six items. Two items were added: respondent type (patient or caregiver), education and employment status. Two dichotomous items replaced the original four general health items: hospitalisation in the preceding 12 months and prior clinical trial participation.

Within the motivators section, two items were merged: weekend visit flexibility and telemedicine access, as both reflect scheduling adaptability. One item was removed; contributing to something important for the general welfare; its content overlaps conceptually with two retained altruism items: contributing to future improvements for other patients with EB, and contributing to increased knowledge about the disease. One item assessing material rewards was also removed. Two open-ended items defining study success and listing further motivators were also removed to improve completion rates.

Within the barriers section, personal financial burden and personal circumstances were merged into one item with a free-text response field; both reflect participation constraints arising from non-medical factors. Two novel barrier items were added: lack of clear study information and uncertainty about medication effectiveness.

The questionnaire was translated into Arabic and underwent linguistic and cultural adaptation. Responses were recorded on a five-point Likert scale, graded from 1 (not at all present/not at all important) to 5 (very high/utmost important). Internal consistency was assessed using Cronbach's alpha. The overall alpha across all 30 Likert-scale items was excellent (*α* = 0.939). The full questionnaire is provided in Supplementary Tables S1, S2.

### Data collection

The questionnaire was distributed electronically to participants via phone or email between March and September 2025. Patients completed the questionnaire independently; primary caregivers completed it on behalf of participants who were unable to do so. The Institutional Review Board at Al- Imam Mohammad Ibn Saud University granted ethical approval (reference: 761/2025), and all participants provided written informed consent prior to enrolment.

### Statistical analysis

Data analysis was performed using IBM SPSS Statistics, version 23 (IBM Corp., Armonk, NY, USA). Categorical variables are presented as frequencies and percentages. Non-normally distributed continuous variables are presented as median and interquartile range. Associations between categorical variables were examined using Fisher's exact test. The Mann–Whitney *U*-test and the Kruskal–Wallis test were applied to compare continuous variables across groups. A *p*-value of less than 0.05 was considered statistically significant.

## Results

### Patient characteristics

A total of 35 participants were enrolled. EB subtypes included DEB in 45.7% (16/35), EBS in 31.4% (11/35), JEB in 14.3% (5/35), and unknown subtype in 8.6% (3/35). The majority of participants were younger than 18 years of age: 68.6% (24/35), while 31.4% (11/35) were aged 18 years or older. Questionnaires were completed by caregivers in 57.1% (20/35) of cases and by patients themselves in 42.9% (15/35). Further demographic data are presented in Table 1.

**Table 1:** Demographic and clinical characteristics of the participants (*n* = 35).

Characteristics	Patients (*n*)	Percentage (%)
Gender		
Male	20	57.10
Female	15	42.90
Age (years)		
Mean	13.5	
Range	1–37	
≥18	11	31.4
<18	24	68.6
Nationality		
Saudi	23	65.71
Non-Saudi	12	34.29
Type of EB		
EBS	11	31.4
JEB	5	14.3
DEB	16	45.7
Unknown	3	8.6
Current educational level		
Pre-school age	8	22.90
Student	18	51.40
Dropped out	3	8.60
Employee	4	11.40
Non-employee	2	5.70
Hospitalization due to EB in the last year		
Yes	13	37.1
No	22	62.9
Prior participation in clinical trials		
Yes	5	14.3
No	30	85.7

EB, epidermolysis bullosa; EBS, epidermolysis bullosa simplex; JEB, junctional epidermolysis bullosa; DEB, dystrophic epidermolysis bullosa.

### General clinical background

Hospitalisation due to EB complications in the preceding 12 months was reported by 37.1% (13/35) of participants, while 62.9% (22/35) did not require hospitalisation. Upon stratification by EB subtype, hospitalisation was more frequent in patients with severe subtypes (JEB/DEB) than those with milder subtype (EBS): 42.9% (9/21) vs. 18.2% (2/11) (*p* = 0.248).

### Knowledge and information about clinical studies

**Figure 1:**
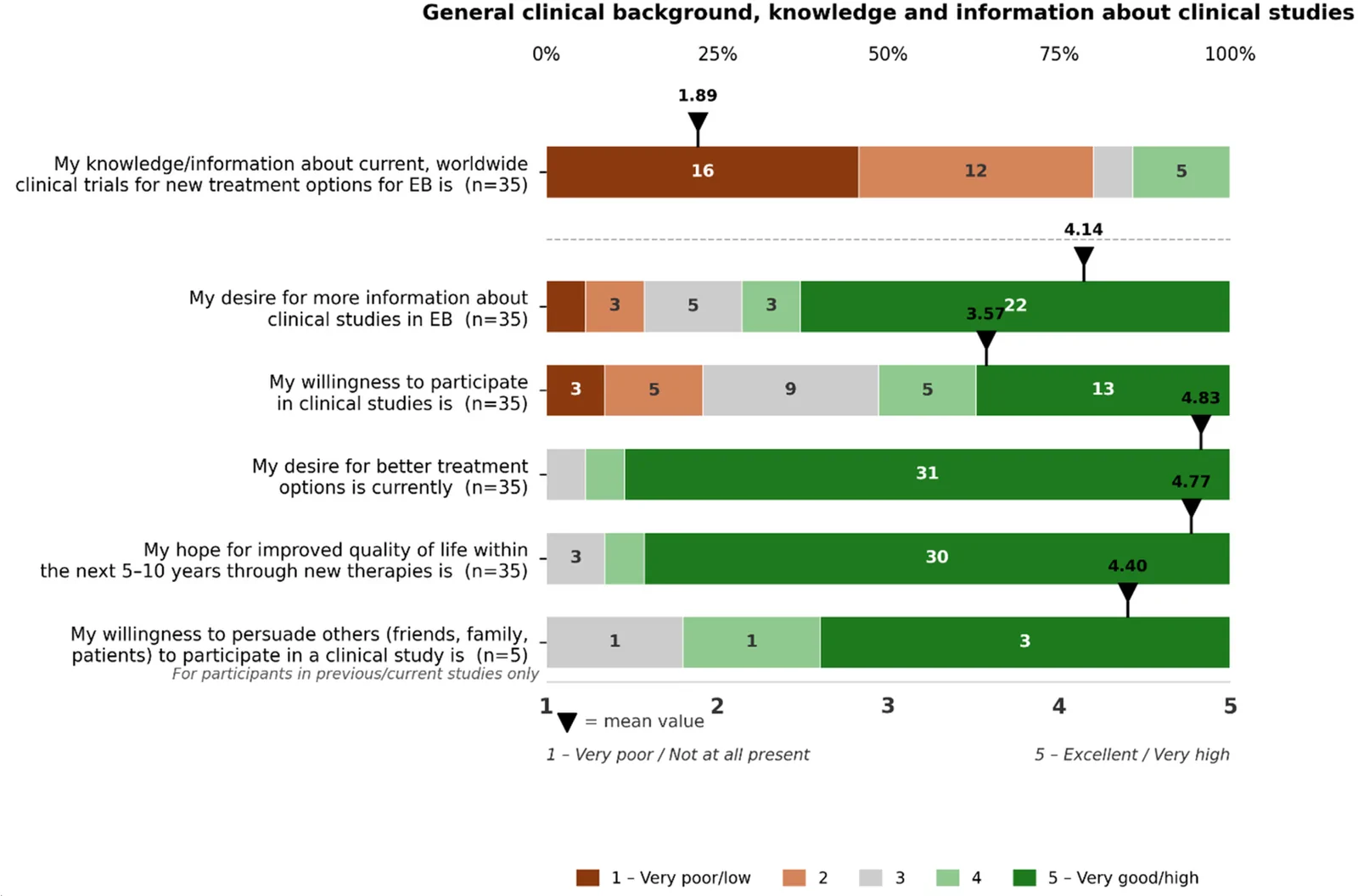
General clinical background, knowledge and information about clinical studies. Graphical presentation of participants' responses (in percentage) to the knowledge and information section of the survey, which includes items addressing self-assessed knowledge about EB clinical trials and research interest. Mean values (Likert scale graded from 1 to 5) are indicated by downward triangles. The numbers in the columns represent the number of respondents for each response option.

Overall knowledge about clinical trials in EB was low; 80% (28/35) of participants reported little or no knowledge. Consequently, the desire to receive more information as well as to increase personal knowledge about current EB research was very high in 62.9% (22/35) of all participants (Figure 1).

**Figure 2:**
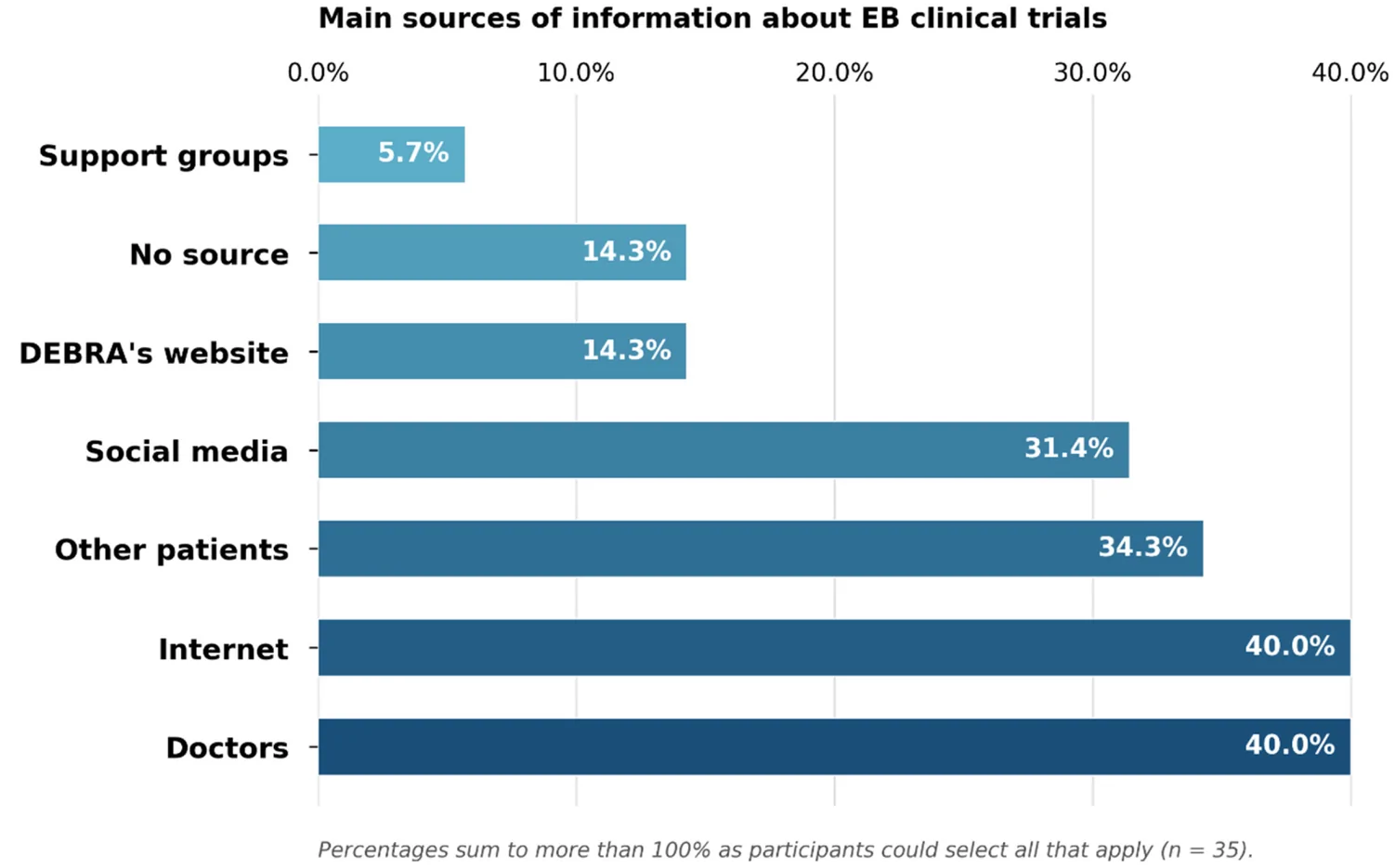
Main sources of information about EB clinical trials. The figure presents the percentage of participants who identified each source. Percentages sum to more than 100% as participants could select all that apply (*n* = 35).

Participants identified doctors and the internet (40% [14/35] each), other patients (34.3% [12/35]), social media (31.4% [11/35]), DEBRA's website (14.3% [5/35]), and support groups (5.7% [2/35]) as their sources of information (Figure 2).

Willingness to participate in a clinical trial was high or very high in 51.4% (18/35) of all participants. Patients with severe subtypes (JEB/DEB) expressed higher willingness than those with milder subtype (EBS): 57.1% (12/21) vs. 36.4% (4/11) (*p* = 0.458). Participation in a clinical trial was favoured by 60.0% (3/5) of participants with prior clinical trial experience compared to 50.0% (15/30) of those without (*p* = 1.000). The level of knowledge was statistically significantly associated with the desire to participate in clinical trials; participants with higher knowledge expressed greater desire than those with little or no knowledge: 85.7% (6/7) vs. 42.9% (12/28) (*p* = 0.001).

Irrespective of EB subtype or age group, the desire for better treatment options was high or very high in 94.3% (33/35) of all participants. Hope for improved quality of life through new therapies within the next 5–10 years was very high in 85.7% (30/35) of all participants; this was more pronounced in patients with severe subtypes (JEB/DEB) than those with milder subtype (EBS): 90.5% (19/21) vs. 72.7% (8/11) (*p* = 0.310).

### Motivators for trial participation

**Figure 3:**
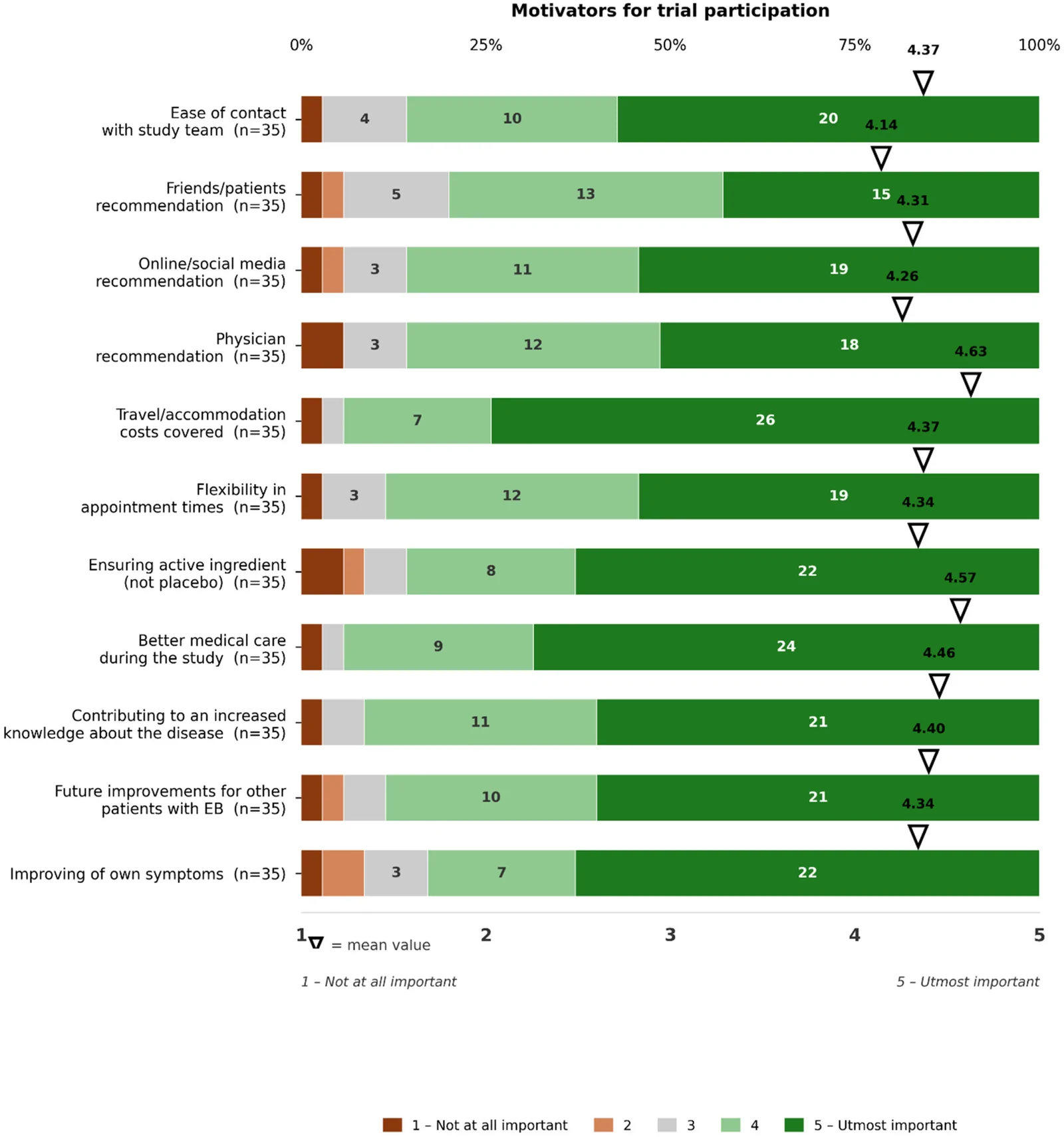
Motivators for trial participation. Graphical representation of participants' responses (in percentage) to the motivators section of the survey, which comprises items addressing the main arguments for participation in a clinical trial. The list follows the order of the questionnaire. Mean values (Likert scale graded from 1 to 5) are indicated by downward triangles. The numbers in the columns represent the number of respondents for each response option.

Practical motivators ranked highest among all participants; travel and accommodation costs being covered and receiving better medical care during the study period were rated as highly important by 94.3% (33/35) each. Altruistic motivators followed closely; contributing to increased knowledge about the disease and future improvements for other patients with EB were rated as highly important by 91.4% (32/35) and 88.6% (31/35) respectively. Symptom alleviation was rated as highly important by a greater proportion of patients with severe subtypes (JEB/DEB) than those with milder subtype (EBS): 85.7% (18/21) vs. 72.7% (8/11) (*p* = 0.390). Among participants with high desire to participate, symptom alleviation was rated as highly important by a greater proportion than those with low or moderate desire: 94.4% (17/18) vs. 70.6% (12/17) (*p* = 0.088) (Figure 3).

### Barriers to trial participation

**Figure 4:**
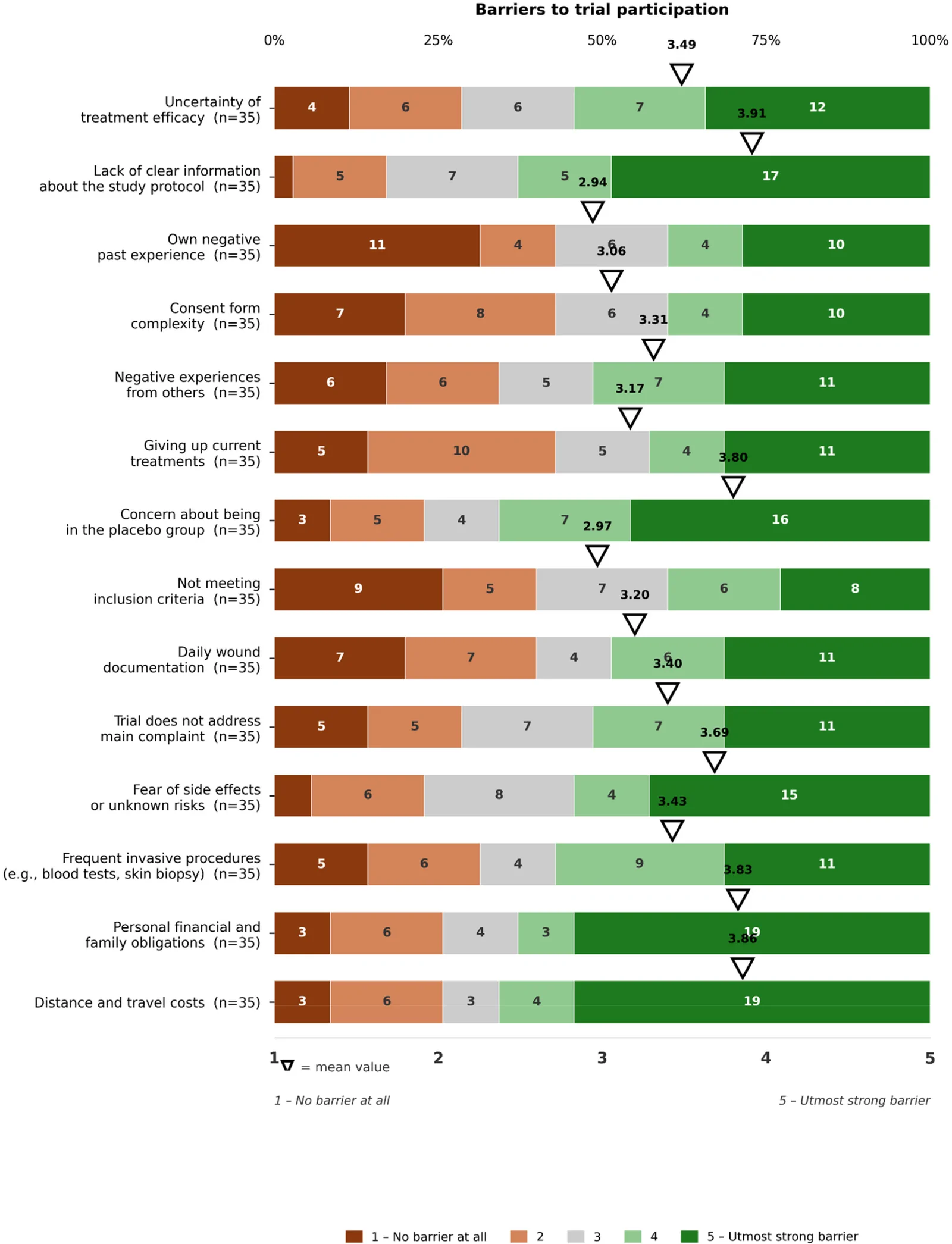
Barriers to trial participation. Graphical representation of participants' responses (in percentage) to the barriers section of the survey, which comprises items addressing the main obstacles to participation in a clinical trial. The list follows the order of the questionnaire. Mean values (Likert scale graded from 1 to 5) are indicated by downward triangles. The numbers in the columns represent the number of respondents for each response option.

Distance to the study site and travel costs and concern about being in the placebo group were the most prominent barriers, each rated as a major or very major obstacle by 65.7% (23/35) of participants. These were followed closely by lack of clear information about the study protocol and personal financial and family obligations, each reported by 62.9% (22/35). Fear of side effects or unknown risks and uncertainty of treatment efficacy were rated as major barriers by 54.3% (19/35) each. Extent and comprehensibility of the informed consent document was rated as a major barrier by 40.0% (14/35) (Figure 4).

Among participants younger than 18 years, frequent invasive procedures tended to be a more prominent barrier than among those aged 18 years and older: 66.7% (16/24) vs. 36.4% (4/11) (*p* = 0.144). Conversely, uncertainty of treatment efficacy tended to be more prominent among older participants: 72.7% (8/11) vs. 45.8% (11/24) (*p* = 0.167).

## Discussion

Our results demonstrated that the level of knowledge was statistically significantly associated with the desire to participate in EB clinical trials (*p* = 0.001); participants with higher knowledge expressed greater desire to participate than those with little or no knowledge: 85.7% (6/7) vs. 42.9% (12/28). Yet knowledge about EB clinical trials was low in 80% of participants, representing a critical gap that directly limits trial participation. This is consistent with evidence from rare disease populations showing that patient education significantly increases trial enrolment (13). Similar patterns have been reported in other Saudi disease populations, where lack of information was identified as a key barrier to trial participation (14). Addressing this knowledge gap through targeted patient education and information strategies is therefore essential to capitalise on the high motivation observed in this cohort.

Despite low knowledge, the desire to receive more information and to participate was high; nearly two-thirds expressed a very high desire for information and more than half expressed high or very high willingness to participate. These findings present a clear opportunity for clinical research in EB in Saudi Arabia. To capitalise on this opportunity, targeted patient education and information strategies are essential. Caregivers of younger patients comprised the majority of our cohort; their high desire for better treatment options and hope for improved quality of life signal strong motivation that can be channelled into trial participation through accessible study information.

Physicians and the internet were the primary sources of information about EB clinical trials (40% each), yet physician recommendation ranked relatively low as a motivator. This apparent discrepancy; physicians are trusted information sources but less decisive as motivators, mirrors findings from multiple clinical trial populations (12, 15). It may reflect two separate dimensions: seeking information through physicians and independent decision-making based on self-acquired knowledge. It may also reflect clinician reluctance toward clinical trials, driven by concerns about placebo allocation, treatment discontinuation, or protocol burden (12). Given that EB is managed across multiple specialties, periodic specialty-targeted education programmes including continuing medical education workshops and EB-specific research training represent an actionable strategy to improve physician engagement with trial referral and enrolment (5–7, 12).

In our cohort, practical motivators ranked highest; the vast majority rated travel and accommodation cost coverage and better medical care during the study period as highly important. Evidence from other patient populations has shown that patients prioritise practical and financial incentives when considering trial participation (16). Altruistic motivators followed closely; contributing to increased knowledge about the disease and future improvements for other patients with EB were rated as highly important by the great majority of participants, consistent with findings across rare disease populations where altruism consistently ranks among the strongest participation drivers (17). This contrasts with the Austrian cohort, where altruism was the primary motivator (12). Symptom alleviation tended to be more important among participants with high desire to participate than those with low or moderate desire, consistent with evidence from other rare disease populations showing that symptom burden is a primary driver of trial motivation among engaged patients (13).

Distance and travel costs and concern about being in the placebo group were the most prominent barriers, each rated as a major obstacle by nearly two-thirds of participants. Travel burden is an inherent challenge in rare disease trials; the geographic dispersion of patients with EB across Saudi Arabia and the absence of a dedicated national EB centre create wide catchment areas that impose substantial travel demands (18). Physical impairments, particularly in patients with severe subtypes, further compound this burden; daily life activities in this subgroup are highly restricted by dressing changes, standard clinical appointments, and caregiver commitments, making additional transportation to a study site a significant deterrent (19, 20).

Placebo concern ranked equally high as a barrier. This finding underscores the need for clear, accessible informed consent processes that accurately explain placebo allocation and the availability of active treatment following trial completion (21). Lack of clear information about the study protocol was rated as a major barrier by nearly two-thirds of participants, directly linking back to the low knowledge identified in our cohort and reinforcing the critical role of transparent patient-facing study materials.

Subgroup analysis showed that patients with severe subtypes (JEB/DEB) had numerically higher willingness to participate than those with milder subtype (EBS): 57.1% vs. 36.4%, likely reflecting a higher medical need and greater urgency for new treatment options. Among younger participants, frequent invasive procedures tended to be a more prominent barrier than among older participants. Given that the majority of our cohort were under 18 years, trial protocols in EB should minimise invasive procedures and incorporate flexible designs; including telemedicine visits and home-based assessments, to the extent scientifically permissible (21, 22). Additional paediatric-specific barriers warrant consideration, including route and frequency of medication administration and school absenteeism; these factors compound the already substantial daily care burden in this age group and should inform the design of future EB trials targeting paediatric populations (19, 20).

This study has several limitations. Recruiting an adequate sample size in a rare disease such as EB is inherently challenging due to the small number of eligible patients; the limited sample size in our study reduced statistical power and may affect the representativeness of the findings. Caregiver proxy-completion by the majority of respondents may introduce reporting bias; caregivers' perspectives may not fully reflect patients' own attitudes. The vast majority of participants had no prior clinical trial experience, which may have introduced optimism bias; responses may reflect idealistic rather than informed attitudes toward participation. Finally, the study assessed hypothetical trial participation rather than actual enrolment, a limitation common to studies of this design.

## Conclusions

This study provides actionable guidance to improve patient-centred trial design in EB and other rare skin diseases and offers characterisation of clinical trial perspectives in this population. Trial recruitment in EB requires targeted education programmes to raise awareness and address placebo misconceptions, and patient-centred designs that minimise travel burden and invasive procedures. The high prioritisation of symptom alleviation as a motivator; particularly among participants with high desire to participate and those with severe subtypes; underscores the importance of incorporating patient-reported outcome measures as primary or co-primary endpoints in future EB trial design, ensuring that the outcomes patients care most about are systematically captured (13, 21). As novel therapies for EB continue to emerge, understanding and addressing the perspectives of patients and caregivers is essential to ensure equitable access to clinical research across the EB community.

## Data Availability

The original contributions presented in the study are included in the article/Supplementary Material, further inquiries can be directed to the corresponding author/s.
