# Supplementary material for: Clinical trial awareness and participation attitudes among patients with epidermolysis bullosa and their caregivers: a nationwide cross-sectional study from Saudi Arabia
Source: Front Med (Lausanne). 2026 Sep 3;13:1913338. doi: 10.3389/fmed.2026.1913338 (PMC13581503; doi:10.3389/fmed.2026.1913338)
Supplement: Supplementary file 1 [file Table1.docx]

**SUPPLEMENTAL TABLE LEGENDS**

**Table S1.** *English version of the questionnaire used in the study*

Modified with permission from Prodinger et al. *Orphanet J Rare Dis.* 2020;15:182.

**DEMOGRAPHICS**

I am completing this questionnaire as: □ Patient □ Caregiver/Parent *(completing on behalf of a minor)*

Nationality and place of residence: _______________________	Age: _________

EB-subtype: _______________________ Gender: _______________________

Education/employment status: _______________________

**GENERAL**

|  | **Yes** | **No** |
| --- | --- | --- |
| Have you been hospitalized in the past year due to EB complications? | □ | □ |
| Have you ever participated in a clinical trial for EB treatment? | □ | □ |

|  | **Very poor** | **Poor** | **Fair** | **Good** | **Excellent** |
| --- | --- | --- | --- | --- | --- |
| My knowledge/information about current, worldwide clinical trials for new treatment options for EB is: | □ | □ | □ | □ | □ |

From where do you retrieve information about current clinical EB studies? *(Select all that apply)*

□ Doctors	□ Local support groups	□ Social media	□ Other patients	□ DEBRA □ Internet	□ I have no source

□ Other (please specify: _____________________________________________)

**INFORMATION ABOUT CLINICAL STUDIES / RESEARCH**

| **For each statement below, you may choose from 5 possible answers. There are no right or wrong answers. Answer as spontaneously as possible while thinking about your situation.** | **Not at all present** | **Low** | **Moderate** | **High** | **Very high** |
| --- | --- | --- | --- | --- | --- |
| My desire for more information about clinical studies in EB | □ | □ | □ | □ | □ |
| My willingness to participate in clinical studies is | □ | □ | □ | □ | □ |
| My desire for better treatment options is currently | □ | □ | □ | □ | □ |
| My hope for improved quality of life within the next 5–10 years through new therapies is | □ | □ | □ | □ | □ |
| *For those who have previously participated in a clinical study:* My willingness to persuade others (friends, family, patients) to participate in a clinical study is | □ | □ | □ | □ | □ |

**Table S2.** *Motivators and barriers to clinical trial participation among patients with epidermolysis bullosa and their caregivers*

**How important are the following reasons for you to participate in a clinical trial?**

| **For each statement below, you may choose from 5 possible answers. There are no right or wrong answers. Answer as spontaneously as possible while thinking about your situation.** | **Not**  **at all important** | **Slightly important** | **Neutral** | **Important** | **Utmost important** |
| --- | --- | --- | --- | --- | --- |
| I want to reach alleviation of my symptoms | □ | □ | □ | □ | □ |
| I want to contribute to future improvements for other EB patients | □ | □ | □ | □ | □ |
| I want to contribute to an increased knowledge about the disease | □ | □ | □ | □ | □ |
| Participation in the study gives me more confidence since I receive better medical care due to increased frequency of study visits | □ | □ | □ | □ | □ |
| If I participate, I want to be sure that I receive treatment with active substance and that I am not in the placebo group (without active substance) | □ | □ | □ | □ | □ |
| Study visits take into account my needs and are flexible (e.g., they can take place on weekends), and can be done via video call/telemedicine from home | □ | □ | □ | □ | □ |
| Participation in the study does not cost me anything (i.e., replacement of travel and food costs) | □ | □ | □ | □ | □ |
| My physician recommends participation | □ | □ | □ | □ | □ |
| Participation in the study is recommended on the internet/social networks/EB forums | □ | □ | □ | □ | □ |
| The study is recommended by friends or other patients | □ | □ | □ | □ | □ |
| A contact person from the study team is constantly available by phone (day and night) | □ | □ | □ | □ | □ |

**What are your main barriers for participating in a clinical trial?**

| **For each statement below, you may choose from 5 possible answers. There are no right or wrong answers. Answer as spontaneously as possible while thinking about your situation.** | **No barrier at all** | **Minor barrier** | **Moderate barrier** | **Strong barrier** | **Utmost strong barrier** |
| --- | --- | --- | --- | --- | --- |
| The distance of the study center / travel expenses | □ | □ | □ | □ | □ |
| Personal financial expenditures or incompatibility with occupational/private obligations or personal circumstances prevent me from participating (please specify: _____________________________) | □ | □ | □ | □ | □ |
| Extent of scheduled invasive investigations (e.g., blood taking, biopsy) | □ | □ | □ | □ | □ |
| Extent of possible adverse reactions or unknown risks of the study medication | □ | □ | □ | □ | □ |
| The expected effect of the study medication does not address the most important complaints of my disease | □ | □ | □ | □ | □ |
| Need to daily document the wound situation and complaints (e.g., study diary) | □ | □ | □ | □ | □ |
| I do not meet the inclusion criteria for the study (e.g., due to small wound area or too many comorbidities) | □ | □ | □ | □ | □ |
| I am worried about being in the placebo group (no active ingredient) | □ | □ | □ | □ | □ |
| I would have to stop my usual treatment (for example, ointments, bandages) during study participation | □ | □ | □ | □ | □ |
| I have heard about negative experiences from other study participants *(please specify: _____________________________)* | □ | □ | □ | □ | □ |
| Extent and comprehensibility of the information materials (including informed consent document) for a study | □ | □ | □ | □ | □ |
| I have made negative experiences in previous studies *(please specify: _____________________________)* | □ | □ | □ | □ | □ |
| Lack of clear information about the study protocol | □ | □ | □ | □ | □ |
| Uncertainty about the study medication effectiveness | □ | □ | □ | □ | □ |

Would you like to tell us anything else about clinical trials?

____________________________________________________________________________________

____________________________________________________________________________________

____________________________________________________________________________________

____________________________________________________________________________________

____________________________________________________________________________________

____________________________________________________________________________________

Modified with permission from: Prodinger C, Diem A, Ude-Schoder K, Piñón-Hofbauer J, Kitzmueller S, Bauer JW, Laimer M. Profiling trial burden and patients’ attitudes to improve clinical research in epidermolysis bullosa. *Orphanet J Rare Dis.* 2020 Jul 10;15(1):182. https://doi.org/10.1186/s13023-020-01443-3

**Thank you for completing this questionnaire!**
